# 

**Published:** 1896-10

**Authors:** 


### PERSONAL.

Dr. Charles M. Cullen, of West Philadelphia, has been nominated to represent the Twenty-fourth Ward in the State Legislature.

Dr. W. S. Manley, of Arkansas City, Kansas, writes that a good opening for a competent veterinarian may be learned of by writing him at the above address.

Veterinarian Waugh, of Pittsburg, is heartily in favor of schools of farriery in large cities.

Prof. V. A. Moore, of the New York State College, was recently a delegate to the American Microscopical Society at Pittsburg, Pa., and was elected by that body as a committee of one to oppose, for that organization, the bill in Congress known as Senate bill No. 1552.

Thomas King, V.S., Principal of the Ohio Veterinary College at Cincinnati, Ohio, is taking a medical course, and expects to graduate in human medicine the coming spring.

Dr. H. J. McClellan, of Lansdowne, Pa., has retired from the practice of veterinary medicine and will enter the field of real-estate investments.

Dr. S. H. Johnson, of Philadelphia, has purchased the good-will and practice of Dr. H. J. McClellan, and will locate permanently at Lansdowne.

Prof. Davenport, Dean of the Illinois Agricultural College, will teach animal husbandry under the new assignments of subjects in that institution.

James Graham, M.R.C.V.S., of Germantown, Pa., is suffering with serious injuries to his back, received through an accident while out riding. His carriage was upset while passing over a pile of cinders.

Veterinarian G. Howard Davison, of Millbrook, N. Y., was a winner of prizes for his show sheep at the Indiana and Wisconsin State Fairs.

Dr. F. L. Kilborne will not be connected with the Veterinary Department of Columbian University this fall.

Veterinarian H. M. Mudge, a recent graduate of the Detroit Veterinary College, will associate a shoeing-forge with his practice at Willis, Mich.

Dr. E. L. Volgenau, formerly of Buffalo, N. Y., having recently passed the civil-service examination, has been appointed to a place in the department and assigned to duty in Chicago.

Dr. James T. McAnulty, of Philadelphia, President of the Pennsylvania State Organization of Master Horseshoers, reports a very successful meeting of the craft at Scranton in September.

Veterinarian W. H. Robinson, of Somerville, Mass., was a visitor, and addressed the members of the Master Horseshoers at Scranton, Pa.

Veterinarian M. D. Williams, of Middleport, N. Y., is getting out a breeders' directory of live stock, poultry, and pet stock.

Dr. J. P. Lowe, of Passaic, N. J., will be a good man to lead the closer affiliation of all the veterinarians of New Jersey in one good, strong association, and then better legislation for the people and a higher recognition for the veterinarian will follow.

Drs. R. M. and John N. Navin, graduates of the American Veterinary College, have retired from practice, and with their brother, Arthur M., are conducting three drug stores in Indianapolis, Ind., under title of Navin's Pharmacy No. 1, No. 2, and No. 3. These young men are sons of the late John N. Navin, Sr., who was a distinguished author and pioneer veterinarian in Indiana.

E. T. Hagyard, Jr., V.S., of Lexington, Kentucky, will succeed Dr. M. E. Knowles as veterinarian on the breeding-farm of Marcus Daly on Bitter Root River, Montana. The salary of $4000 is said to be still continued.

Our good friend, Veterinarian N. Rectenwald, of Pittsburg, rejoices in the happy feelings of being a grandfather. Hence his added joys at Buffalo.

The Allegheny County Sanitary Association, composed of physicians and veterinarians, meets monthly in Pittsburg, Pa.

Prof. W. Williams has been sent to the Island of Jamaica to investigate an outbreak of disease among the cattle, which has already caused great losses.

Dr. F. O. Richmond occupies the post of secretary and veterinarian to the Live-stock Sanitary Commission for the Territory of Arizona.

David Waugh, a registered practitioner of Western Pennsylvania, and who heretofore has largely devoted his time to castration of cryptorchids, will enter the Veterinary College at Indianapolis.

The veterinarians to the National Horse Show at Madison Square Garden for the coming exhibition, November 9th to 14th, inclusive, are: Drs. Wm. Sheppard, Edward Loomes, and Thomas G. Sherwood, all of New York.

Veterinarian W. H. Dalrymple, of Baton Rouge, La., is editor of the Agricultural Department of the *Weekly Picayune*, published in New Orleans. His department is brimful of interesting matter regarding live-stock, and his pen is wielded with considerable force for the advancement of science and skill by those whose chief interest is in the development of the highest grade of domestic animals.

Dr. J. O. Reed, of Danville, Pa., was a recent visitor to the *Journal* office. He purposes entering the fall examination by the Pennsylvania State Board of Veterinary Medical Examiners.

---

Mr. M. W. Rudderow, of Philadelphia, a breeder of hackneys, reports a singular freak following an accident to an imported mare bred to the hackney stallion Hopeful at Mr. Walter C. Clark's farm in Virginia. When about four months' pregnant the barn was fired, and this mare, in escaping, was caught in a gate, breaking a rib on each side; she was also scorched about the limbs and body. The broken ribs were followed by depression of the parts injured, which, in healing, left a large lime-salt deposit at the points of continuity. The colt when foaled had two similar depressions and enlargements identical with the mare, and rough places, gray in color, in the body and limbs, corresponding with the burnt areas of the dam. The colt, a chestnut, now two years old, retains the depressions, which mar its appearance very much; but the areas of gray hair and thickened skin have disappeared.

